# Imaging in neuroblastoma

**DOI:** 10.1007/s00247-022-05489-2

**Published:** 2022-09-05

**Authors:** Annemieke S. Littooij, Bart de Keizer

**Affiliations:** Department of Radiology and Nuclear Medicine, University Medical Center Utrecht, Heidelberglaan 100, 3584 CX Utrecht, The Netherlands

**Keywords:** Children, Imaging, Magnetic resonance imaging, Neuroblastoma, Nuclear medicine

## Abstract

Neuroblastoma is the most common extracranial solid malignancy of childhood. The prognosis is highly variable ranging from spontaneous involution in infants to fatal outcome, despite aggressive treatment, in disseminated high-risk neuroblastoma. This paper provides a comprehensive review of the crucial role of imaging during the extensive treatment course.

## Introduction

Neuroblastoma is the most common and deadly extracranial solid malignancy occurring during childhood. However, the incidence is low, with approximately 10 cases per million children under 15 years of age [1]. Neuroblastoma originates from the peripheral sympathetic nervous system. About half of the tumours arise from the adrenal gland. Other sites of origin are the organ of Zuckerkandl or along the paravertebral ganglia from neck to the pelvis [1, 2].

Neuroblastoma is characterized by a broad spectrum of clinical behaviour, which can range from spontaneous regression without treatment, to maturation into a benign ganglioneuroma or widespread disseminated disease that is unresponsive to aggressive treatment [1–3]. In order to provide the optimal treatment to such variedly behaving disease, there are stratifying criteria to define risk groups. This risk stratification is dependent on the age of the child, stage of disease, histopathological results, status of the MYCN oncogene, DNA ploidy, aberrations of chromosomes and other biological factors [3]. Tumours with MYCN amplification are all considered high risk whether localized or disseminated. Approximately half of affected children are considered high-risk patients. Treatment of high-risk neuroblastoma consists of a combination of chemotherapy, surgery, radiotherapy, stem cell transplant and immunotherapy [4].

Multimodality imaging including metabolic nuclear imaging are essential components for diagnosis, staging, response assessment and follow-up.

## Clinical presentation

The clinical presentation is highly variable and depends on the site of origin and the presence of bone marrow dissemination or paraneoplastic syndromes. Three main clinical presentations are commonly encountered: localized tumours, disseminated disease and 4S/MS disease [1, 2].

Around 40% of patients present with localized disease. This can range from antenatally detected adrenal tumours that tend to regress spontaneously to symptomatic paraspinal neuroblastoma. Patients presenting with a paraneoplastic syndrome often have localized tumours. Opsoclonus-myoclonus syndrome is the most often encountered paraneoplastic syndrome and is seen in 2–4% of patients with neuroblastoma. It is a neurological disorder characterized by rapid, multidirectional eye movements, quick, involuntary muscle jerks and ataxia. Although patients with opsoclonus-myoclonus syndrome have good prognosis regarding their tumour, the majority will have long-term neurological deficit [5].

**Fig. 1:**
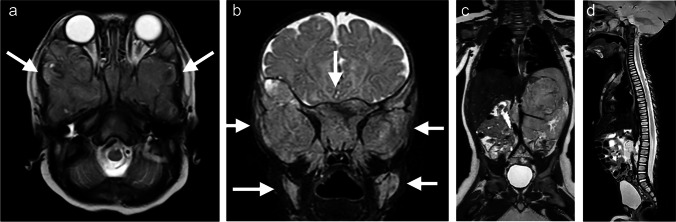
A 5-month-old boy with raccoon eyes (periorbital ecchymosis) due to extensive bone marrow metastasis from abdominal neuroblastoma. An axial T2-weighted image (**a**) and a coronal short tau inversion recovery (STIR) image (**b**) of the skull base show extensive bone marrow metastases around both orbits (*arrows*). **c** A coronal T2-weighted image shows the primary tumour arising from the left adrenal gland with vascular encasement of the renal pedicle (Image Defined Risk Factors positive). **d** A sagittal STIR image illustrates diffuse bone marrow metastasis of the spine

Around 50% of patients present with metastatic neuroblastoma (Fig. 1). These children are typically ill at presentation. In cases of widespread bone marrow disease, patients often present with bone pain, limping or irritability. Additionally, symptoms of bone marrow failure can occur.

**Fig. 2:**
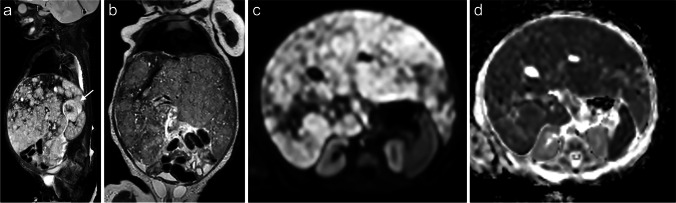
A 1-month-old girl with enlarged abdominal girth, thrombocytopenia and anaemia. A sagittal short tau inversion recovery (STIR) image (**a**) shows the relatively small primary tumour arising from the right adrenal gland (*arrow*) with diffuse liver metastases. **b** A coronal T2-weighted image illustrates enlargement of the liver due to diffuse liver metastases. Treatment was started due to respiratory compromise. The liver metastases show impeded diffusion (**c**: axial b1,000 diffusion-weighted image; **d**: axial apparent diffusion coefficient map)

Stage MS or 4S is reserved for patients younger than 18 months with metastases confined to liver, skin and bone marrow. However, bone marrow metastases should be limited to less than 10% on smears or biopsy. Typically, these infants present with a relatively small primary tumour with widespread metastatic disease. Rapidly progressive intrahepatic involvement can result in respiratory failure and necessitates treatment (Fig. 2), while some tumours will need no treatment and disappear spontaneously [6].

## Diagnosing and staging neuroblastoma

Ultrasound (US) is often the first-line modality to detect neuroblastoma particularly when located in the neck, abdomen or pelvis. Ultrasound features that suggest neuroblastoma are internal calcifications and encasement of the vessels. Often, bulky lymphadenopathy is seen. Neuroblastoma can extend through the neuroforamina into the spinal canal. Further cross-sectional imaging is required to accurately assess the extent of disease [7].

Although computed tomography (CT) is an excellent modality, magnetic resonance imaging (MRI) is preferred, if available, due to its intrinsic high contrast and radiation-free images and its capability to provide additional functional information about the tumour. MRI is superior to CT in assessing bone marrow disease and chest wall invasion and should be the default imaging in cases of spinal canal involvement. Diffusion-weighted imaging (DWI) is especially useful for neuroblastoma as these tumours show strong diffusion restriction. Therefore, detection of distant spread is more easily and more reliably performed with DWI (Table 1). Furthermore, DWI can provide information about heterogeneity of the tumour and thus provide guidance for targeted biopsy in cases with a large mass. Additionally, DWI can be used to identify more differentiated neurogenic tumours, such as ganglioneuroma. These ganglioneuroma lesions show significant higher apparent diffusion coefficient values as compared to poorly differentiated neuroblastoma [8].

**Table 1:** An example of magnetic resonance imaging protocol

Sequence	T2-W STIR	T2-W STIR	DWI^a^	T2-W	T1-W FS	CeT1-W FS
Orientation	Coronal	Sagittal	Axial	Axial	Axial	Axial
Respiratory motion compensation	Respiratory triggering (thorax and abdomen)	Free breathing	Free breathing	Respiratory triggering (thorax/abdomen)	Breath hold	Breath hold
Anatomical coverage	Whole-body	Spine	Whole-body or affected regions	Head to groin	Head to groin	Head to groin

*ceT1-W* contrast-enhanced T1-weighted, *DWI* diffusion-weighted imaging, *FS* fat saturation, *STIR* short tau inversion recovery, *T1-W* T1-weighted, *T2-W* T2 weighted

^a^b0 and b800 or b1,000

Two staging systems exist for patients with neuroblastoma. Since 1986, the International Neuroblastoma Staging System (INSS), basically a post-surgery staging system, has been used. This system therefore depends somewhat on the expertise of the surgical team. In 2009, the International Neuroblastoma Risk Group (INRG) proposed a new staging system (INRGSS) that was designed as a consistent preoperative staging system with imaging as a major component [9]. To enable standardized reporting, Image Defined Risk Factors (IDRFs) were developed to describe the relationship of a tumour with vital structures associated with a high risk of surgical complications (major vessels, nerves and airways) (Table 2) [10].

**Table 2:** International Neuroblastoma Risk Group Staging System^a^

Stage	Description
L1	Localised tumour limited to one body compartment with no IDRFs^b^
L2	Locoregional tumor with one or more IDRFs
M	Distant metastatic disease (except stage MS)
MS	Metastatic disease in children younger than 18 months with metastases confined to skin, liver and/or bone marrow

^a^Adapted from Monclair et al. [9]

^b^*IDRF* Image defined risk factors (a detailed list of which can be found in Brisse et al. [10])

Nuclear medicine studies are used to assess bone marrow metastases with meta-[^123^I] iodobenzylguanidine (MIBG) scintigraphy the most used study. More than 90% of (primary) neuroblastoma are MIBG-avid. MIBG scintigraphy provides two-dimensional (2-D) planar images that can be supplemented with 3-D single-photon emission computed tomography (SPECT) providing combined functional and morphological imaging. Other positron emission tomography (PET) tracers that have the advantage of higher resolution images and shorter scan times such as ^68^gallium Dotatate peptides, ^18^fluorodopa and ^18^fluoro-meta-fluorobenzylguanidine are being studied [11].

## Response assessment

Within Europe, trials on neuroblastoma are conducted by the International Society of Paediatric Oncology (SIOPEN). Within the current High Risk – Neuroblastoma (HR-NBL2) protocol, cross-sectional imaging for response assessment of the primary tumour is performed at staging, end of induction chemotherapy, for preoperative planning, for evaluation of residual primary tumour after surgery and before radiation therapy, before maintenance and at the end of treatment. In anticipation of resection, the relationship of the mass with adjacent organs and vessels should be reassessed for IDRFs, according to the criteria described by Brisse et al. [10]. The IDRFs have proven to be valuable in predicting surgical risk and outcome. [12]

The international neuroblastoma response criteria include the use of RECIST (Response Evaluation Criteria in Solid Tumours) guidance for measurable soft-tissue disease combined with nuclear medicine imaging [13].

The response of bone marrow metastases is assessed with nuclear medicine studies, most often with MIBG scintigraphy. In studies performed by SIOPEN, the SIOPEN score is used where the body is divided in 12 skeletal segments and each segment is assessed for disease with a score of 0 indicating no bone marrow involvement and a score of 6 for diffuse neuroblastoma infiltration of the entire segment. The SIOPEN score has prognostic implications as patients with a score of >3 after induction chemotherapy have very poor outcomes [14]. In case of MIBG-negative bone marrow lesions, [F-18]2-fluoro-2-deoxyglucose (FDG) positron emission tomography (PET)/CT is recommended as an alternative modality for bone marrow assessment.

## Preoperative assessment

Contrast CT scan immediately before surgery is increasingly used as MRI can often underestimate disease extent due to extensive preoperative treatment. The treatment-induced changes, such as fibrosis and calcifications, result in low T1 and T2 signal at MRI, which renders them less noticeable than at diagnosis. Furthermore, the relationship with the venous and arterial vasculature is better appreciated with CT. Therefore, CT is thought to be superior for surgical planning [15]. Another benefit of CT is rapid image acquisition, such that sedation can often be omitted. Presurgical CT scans are optimal when performed as a split bolus to optimise imaging with relation to both arterial/venous anatomy. Furthermore, CT scanning presurgery is particularly important in posterior mediastinal mass lesions (T8-L1) to reliably identify the artery of Adamkiewicz in order to prevent spinal cord ischemia during surgery.

## Assessment of residual disease for radiotherapy planning

Within the current HR-NBL2 protocol, all patients will receive irradiation of the preoperative tumour bed up to 21.6 Gy. Patients with macroscopic residual lesions after induction chemotherapy and surgery will be randomized to receive an additional boost to this residual tumour [4]. However, the imaging criteria for diagnosing macroscopic residual disease are not yet defined and the optimal standardized imaging guidelines not yet implemented. MRI should be preferred to CT when assessing possible residual tumour, due to its better tissue characterization. The MRI should be performed at least 15 days after surgery to avoid misleading findings due to postoperative change. The combination of MRI and MIBG/SPECT is essential. All initial involved soft-tissue lesions should be considered residual tumours. The same applies for previously involved lymph nodes. Other lymph nodes are most likely to be reactive to recent operation or inflammation. Size criteria of >10 mm short axis can be used to identify involved nodes together with a round shape and/or heterogeneous signal intensity. However, these criteria are based on expert opinion and warrant research to determine their accuracy.

## Future/emerging techniques

With the introduction of integrated PET/MRI, combined anatomical, functional and molecular imaging is feasible. This could potentially improve the diagnostic accuracy in assessing bone marrow response in neuroblastoma. Improved detection of intra-tumour heterogenicity might also be achieved.

Radiomics is an emerging field of advanced analysis that aims to extract multiple features from imaging data to provide clinically valuable information. A few recent papers describe the role of CT-based radiogenomics to predict MYCN amplification in patients presenting with neuroblastoma. MYCN amplification is a crucial prognostic factor and is associated with poor survival. Combined with clinical factors (INRGSS stage and extension across the midline) an area under the curve from 0.82 to 0.95 has been reported [16].

## Conclusion

Neuroblastoma is a relatively common paediatric abdominal malignancy. Imaging plays a crucial role in diagnosis, staging, response assessment and follow-up. Except for surgical planning, MRI is the preferred cross-sectional imaging modality for imaging the primary tumour, especially in cases of spinal canal invasion. Nuclear medicine studies are essential for assessing bone marrow disease.
